# Systems biology approach to functionally assess the *Clostridioides difficile* pangenome reveals genetic diversity with discriminatory power

**DOI:** 10.1073/pnas.2119396119

**Published:** 2022-04-27

**Authors:** Charles J. Norsigian, Heather A. Danhof, Colleen K. Brand, Firas S. Midani, Jared T. Broddrick, Tor C. Savidge, Robert A. Britton, Bernhard O. Palsson, Jennifer K. Spinler, Jonathan M. Monk

**Affiliations:** ^a^Department of Bioengineering, University of California San Diego, La Jolla, CA 92039;; ^b^Department of Molecular Virology and Microbiology, Baylor College of Medicine, Houston, TX 77030;; ^c^Alkek Center for Metagenomics and Microbiome Research, Baylor College of Medicine, Houston, TX 77030;; ^d^Space Biosciences Research Branch, Space Biosciences Division, NASA Ames Research Center, Moffett Field, CA 94035;; ^e^Department of Pathology and Immunology, Baylor College of Medicine, Houston, TX 77030

**Keywords:** systems biology, pangenomics, *Clostridioides difficile*, strain typing, metabolic profile

## Abstract

Combatting *Clostridioides difficile* infections, a dominant cause of hospital-associated infections with incidence and resulting deaths increasing worldwide, is complicated by the frequent emergence of new virulent strains. Here, we employ whole-genome sequencing, high-throughput phenotypic screenings, and genome-scale models of metabolism to evaluate the genetic diversity of 451 strains of *C. difficile*. Constructing the *C. difficile* pangenome based on this set revealed 9,924 distinct gene clusters, of which 2,899 (29%) are defined as core, 2,968 (30%) are defined as unique, and the remaining 4,057 (41%) are defined as accessory. We develop a strain typing method, sequence typing by accessory genome (STAG), that identifies 176 genetically distinct groups of strains and allows for explicit interrogation of accessory gene content. Thirty-five strains representative of the overall set were experimentally profiled on 95 different nutrient sources, revealing 26 distinct growth profiles and unique nutrient preferences; 451 strain-specific genome scale models of metabolism were constructed, allowing us to computationally probe phenotypic diversity in 28,864 unique conditions. The models create a mechanistic link between the observed phenotypes and strain-specific genetic differences and exhibit an ability to correctly predict growth in 76% of measured cases. The typing and model predictions are used to identify and contextualize discriminating genetic features and phenotypes that may contribute to the emergence of new problematic strains.

## Significance

*Clostridioides difficile* infections are the most common source of hospital-acquired infections and are responsible for an extensive burden on the health care system. Strains of the *C. difficile* species comprise diverse lineages and demonstrate genome variability, with advantageous trait acquisition driving the emergence of endemic lineages. Here, we present a systems biology analysis of *C. difficile* that evaluates strain-specific genotypes and phenotypes to investigate the overall diversity of the species. We develop a strain typing method based on similarity of accessory genomes to identify and contextualize genetic loci capable of discriminating between strain groups.

The bacterial pathogen *Clostridioides difficile* remains the most common health care–associated infection with an ever-evolving and complex epidemiology. *C. difficile* is recognized as an urgent threat by the Centers for Disease Control and Prevention (CDC) and has been conservatively estimated at over 220,000 cases in hospitalized patients and nearly 13,000 deaths within the United States annually (1). The disruption of natural colonic microbiota following antibiotic use is the leading risk factor for *C. difficile* infection (CDI), and recurrent infections occur in ∼35% of patients (2–4). Two toxins, TcdA and TcdB, are the primary virulence factors for symptomatic infection (5). However, virulence is also attributed by other factors, including the cytolethal distending toxin, sporulation, flagella, and adhesins (6–12). Overall, the plasticity of the *C. difficile* genome has contributed to divergent lineages distinguished by evolutionarily advantageous genetic traits that result in increased antimicrobial resistance, virulence, and metabolic capabilities for survival within the gut (13, 14). The bevy of accessory gene content present across strains in this species has complicated attempts to contextualize strain relationships among this complex population.

Molecular typing techniques that evaluate strain relatedness have been used to evaluate *C. difficile* epidemiology and track transmission of virulent lineages. The *C. difficile* genome has sufficient intraspecies diversity within the intergenic spacer regions of ribosomal RNA (rRNA) genes for the successful use and adoption of PCR ribotyping, the primary molecular typing method for *C. difficile* (15–18). As a result, the most prevalent and hypervirulent *C. difficile* strains globally have been dubbed ribotype (RT) 027 (RT027) and RT078 (12, 19, 20). Additionally, multilocus sequence typing (MLST) is widely used in population studies as a means of distinguishing strains through the allelic profile of designated housekeeping genes (21–23). In addition to these two techniques, there are several other typing methods, including multilocus variable-number tandem repeat analysis, pulsed-field gel electrophoresis, restriction endonuclease analysis, toxinotyping, and surface-layer protein A–encoding gene typing. Each of these methods has unique levels of discriminatory power as well as unique limitations (24). While these typing schemes have proven useful in understanding CDI epidemiology, the most widely adopted schemes (PCR ribotyping and MLST) lack the resolution to distinguish more closely related strains. To obtain mechanistic insight into outbreaks, whole-genome sequencing (WGS) methods need to be employed.

Advancements in sequencing technologies have resulted in an explosion in the availability of quality WGS data (25) promising new and comprehensive approaches to strain typing (26–28). In this age of high-throughput sequencing, comparative genomics analysis has been largely stratified into two approaches: single-nucleotide variants and gene by gene comparisons. In the latter case for *C. difficile*, core-genome multilocus sequence typing (cgMLST) and whole-genome MLST extensions of classical MLST have been developed (29, 30). While these techniques have increased the resolution of typing approaches, key connections between the genomic diversity driving strain types and resulting diversity of phenotypes have remained elusive. A deeper understanding of the functional diversity across this species is needed and must be rooted to the enormous genetic diversity observed.

In recent years, systems biology tools have been challenged with extracting knowledge from the enormous amount of omics data available. In particular, the substantial variability in genomic content and function across strains of a species can be analyzed efficiently through a combination of comparative genomics and various modeling frameworks (31–33). Strain-specific genetic variation can be usefully organized through a pangenomic perspective that delineates and organizes a species’ gene portfolio (34, 35). Additionally, genome-scale models (GEMs) of metabolism have served as tools to mechanistically link genotype to phenotype particularly in terms of growth capabilities. Computation of catabolic capabilities based on genome sequences has provided additional insight into metabolic variability and association to lifestyle niche (36, 37). To increase understanding of the diversity exhibited by *C. difficile*, we have executed a holistic systems biology analysis encompassing both a functional genomics assessment of the pangenome and an in-depth analysis of experimental growth phenotypes aided by construction and use of GEMs. Moreover, we developed a strain typing method based on the accessory gene content, sequence typing by accessory genome (STAG), that allows for explicit investigation into the gene clusters driving the separation of strain groups. This method expands the tool kit for analysis of WGS strain typing across a broad array of disciplines.

## Results

### High-Throughput Phenotypic Screening of *C. difficile* Clinical Isolates Reveals Unique Dynamic Growth Profiles.

**Fig. 1.**
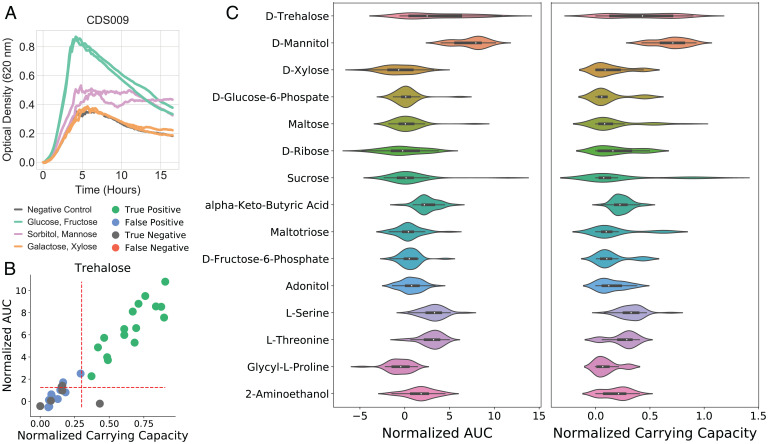
Growth dynamics of *C. difficile* isolates and parameters calculated through GP regression. (*A*) Growth curves for one isolate, CDS009, on 6 of 95 carbon substrates, demonstrating variable growth dynamics and shape of growth curves. (*B*) Each of the 35 isolates’ growth on trehalose plotted in AUC and K with thresholds of 1.25 and 0.3 shown as red dashed lines; strains are colored by corresponding GEM prediction of growth with experimental data. (*C*) Of the 28 discriminatory carbon sources, the top 15 in terms of coefficient of variation of AUC and K between strains are pictured.

To evaluate the metabolic capabilities of *C. difficile*, we profiled 35 clinical strains isolated from hospitalized adult patients using Biolog Phenotype Microarrays and evaluated their ability to catabolize 95 unique carbon sources (*Methods* and https://figshare.com/articles/dataset/Dataset_6_Biolog_Time_Series_Data/19319897). Analysis of the time-course data demonstrated various growth modalities (Fig. 1*A*). Gaussian process (GP) regression models were employed to robustly explore these dynamics (*Methods*). Inferring growth curves and their time derivatives from our data enables the calculation of traditional growth model parameters, such as carrying capacity (K), maximum growth rate, doubling time, and area under the curve (AUC) through a nonparametric approach (Fig. 1*B* and *SI Appendix*) (38). GP regression is advantageous because it has been shown to outperform parametric approaches when considering nontraditional growth-curve shapes, such as diauxic shifts and long lag phases (39, 40).

Overall, unanimous growth determinations (either growth supporting or nongrowth supporting) could be made for 67 compounds, 4 (glucose, fructose, mannitol, *n*-acetyl-d-glucosamine) of which were universally growth supporting across the 35 strains, while the remaining 63 were unanimous nongrowth supporting. The remaining 28 carbon sources assayed support growth in a range of 1 to 34 strains. Therefore, these 28 carbon sources could be used to construct an overall metabolic profile encompassing the growth capabilities on each of these substrates (*SI Appendix*, Fig. S1). For example, CDS031 was the only strain found to grow on galactose, while growth on sucrose was limited to strains CDS071 and CDS031. Niche growth capabilities are identified by examining the outliers in parameter values from the overall set (Fig. 1*C*). In particular, the degree of growth support can be investigated through the calculated AUC and K. Ranking calculated AUC and K reveals which substrates are the strongest strain-specific growth supporters. Outside of the four universal growth-supporting nutrients, the next top five substrates vary across the strains and include mannose, sorbitol, trehalose, sucrose, maltose, glycerol, *n-*acetyl-d-mannosamine, serine, and threonine (*SI Appendix*, Fig. S2). These data indicate that while serine supports growth of multiple strains, only CDS078 grows robustly on serine as one of its best substrates.

### GEM-Predicted Capabilities Capture Discriminatory Metabolic Profiles.

**Fig. 2.**
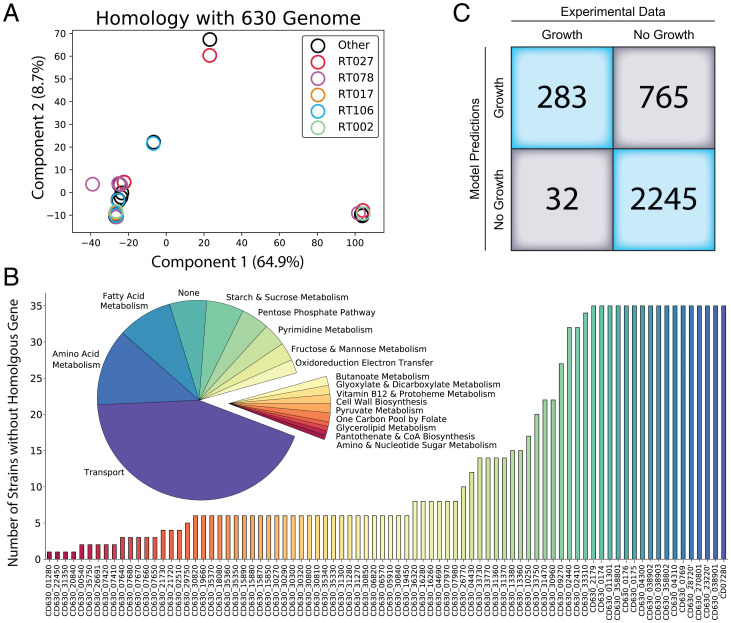
Whole-genome similarity to reference strain 630, deviation in the portion of the gene portfolio contained within iCN900, and overall accuracy of 35 strain-specific models. (*A*) Principal component analysis of the matrix of whole-genome homology of each isolate against *C. difficile* 630. Epidemic ribotypes are highlighted and represented in each cluster, suggesting that their relationship to the reference strain is diverse across these lineages. (*B*) Initial gene content removed from the set of 35 models based on lack of homologous genes from iCN900 and corresponding reaction metabolic subsystems. (*C*) Final agreement of curated strain-specific isolate models and experimental profiling data, resulting in a 76% accurate set of 35 models. CoA, Coenzyme A.

Motivated by the diverse catabolic capabilities identified through our metabolic profiling and subsequent GP regression modeling, we sought to identify the genetic bases for these different capabilities. GEMs, in particular multistrain modeling, provide a powerful tool to contextualize genetic differences and generate metabolic predictions (36, 37, 41–43). Therefore, we generated strain-specific GEMs for each 1 of our 35 isolates based on iCN900, a gold standard reconstruction of *C. difficile* strain 630 (44, 45). To facilitate generating GEMs of our 35 strains, we completed WGS of each isolate (https://figshare.com/articles/dataset/Dataset_7_Isolate_Genomes_zip/19319903) and then, executed a standard protocol to build draft strain-specific models based on the reference reconstruction iCN900 (46). Our preliminary comparative genomics analyses using the reference 630 sequence (AM180355.1) are summarized through principal component analysis of shared genes across the entire genomes of our 35 strains (Fig. 2*A*). This analysis demonstrates that the clinical isolates exhibit variations in conserved genes relative to the reference sequence and that this variation is not consistent across ribotypes.

We evaluated the conserved subsystems of metabolism across the models and found that transport functions, metabolism of particular amino acids, fatty acid metabolism, and starch and sucrose metabolism were most divergent against the reference among the strains (Fig. 2*B*). Specifically, the reactions of phosphotransferase system and adenosine triphosphate binding cassette variety (ABC) system transporters (86%), starch and sucrose metabolism (57%), fatty acid biosynthesis (21%), and lysine and arginine pathways (20.8%) have a high proportion of reactions whose encoding genes contain at least one nonconserved gene (*SI Appendix*, Fig. S3). A major power of GEMs is their ability to predict phenotypes based on the structure of the metabolic network using flux balance analysis (FBA) (47, 48). Thus, we used our strain-specific GEMs to generate model predictions for growth on all 95 carbon sources contained within the phenotypic microarray growth data. In silico growth predictions were generated using previously defined minimal media conditions and alternating the carbon source (*Methods*). Each strain-specific model (https://figshare.com/articles/dataset/Dataset8_CuratedStrainSpecificModels_zip/19319900) was subsequently individually gap filled, and specific false-negative model predictions offered opportunities for further curation (*Methods*). This led to the addition of reactions to specific strains that enabled in silico biomass production for growth on the sole carbon sources pyruvate, *n*-acetyl-d-mannosamine, d-fructose-6-phosphate, d-glucose-6-phosphate, d-serine, and maltotriose, bringing these compounds into agreement with experimental profiles.

Critically, we compared the resulting confusion matrix between our processed experimental dataset and GEM model predictions (Fig. 2*C*), resulting in an overall accuracy of 76% and 0.41 Matthews Correlation Coefficient. Among the incorrect predictions, there were 765 false-positive GEM predictions (Fig. 2*C*), which usually occur because FBA simulation will find any theoretical solution possible dependent on network content and does not consider transcriptional regulation or enzyme efficiency (49). This predictive failure mode in our set of models suggests that in addition to metabolic network diversity, other biological processes play a role in the diverse capabilities of these strains. Thus, we expanded our analysis from curated reactomes to a full pangenome-level analysis.

### Characterization of the *C. difficile* Pangenome Demonstrates Differences in Conservation Based on Functional Classification.

**Fig. 3.**
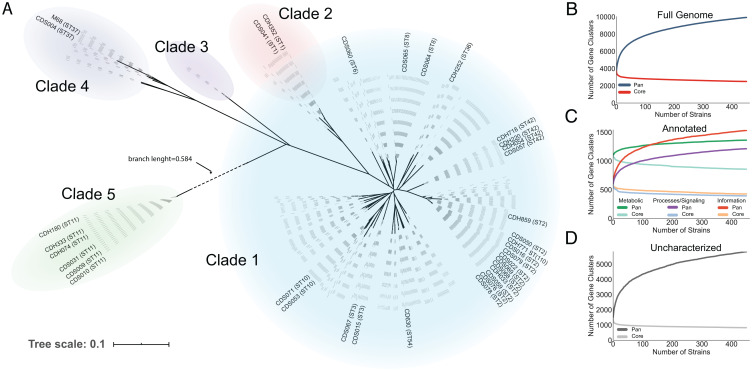
Phylogenomics and pangenome and core-genome curves for the 451 strain set. (*A*) Phylogenomic tree constructed using 451 strains and clinical isolates labeled therein. Each dashed line represents one strain. (*B*) Considering the totality of gene clusters, the core genome is defined by the universally present 2,899 gene clusters, and the remaining 7,025 gene clusters (accessory and unique) make up the rest of the pangenome. (*C*) For gene clusters where functional annotation can be assigned through COG categories, we analyzed the accessory/core breakdown of each major functionally defined group and the behavior of the pangenome curves. (*D*) The uncharacterized or annotated as “function unknown'' clusters make up 68.37% of all gene clusters, and these clusters exhibit the most open behavior in the pangenome curve. This is indicative of the vast number of *C. difficile* genes whose function remains unknown and presents numerous candidates for discovery.

To comprehensively analyze the diversity of strain-specific gene portfolios on a species level, we collected 416 high-quality publicly available genomes (*SI Appendix* and Dataset S2). Along with our clinical isolate dataset, this expanded our overall scope to 451 strains, which were all reannotated to avoid potential biases from differential gene calling (*Methods*). We generated a phylogenomic tree for this dataset and examined how our clinical isolate genomes relate to the public dataset (Fig. 3*A*). Our isolates cover 14 of the 33 major tree branches and thus, span ∼42% of the *C. difficile* phylogeny analyzed here.

To evaluate conserved and unique genes across the strains, we constructed a pangenome using the 451 genome sequences described above (*Methods*). The pangenome is built through efficient all by all sequence homology comparisons that establish gene clusters ranging from unique to ubiquitous genes. Our analysis identified a total of 9,924 gene clusters in the *C. difficile* pangenome, where 2,899 are shared by 99% or more (446 of 451) of the strains and comprise the core genome (Fig. 3*B*). Likewise, we identified 2,968 gene clusters present in only 1% or less (4 of 451) of the strains defining the unique genome. The remaining 4,057 gene clusters represent the accessory genome that is variably present within the population but not present at either the core or unique extremes and therefore, provide a genetic bank rich in discriminatory power.

The gene clusters were functionally annotated using EggNOG (50), and the results were parsed into the broad category Clusters of Orthologous Groups (COGs): metabolism, cellular processes and signaling, and information storage and processing (Fig. 3*C*). Any COG assignments falling under “poorly characterized” were lumped into the genes with no annotation information to form the “uncharacterized” group (Fig. 3*D*). Splitting the pangenome into its functional constituents showed that genes with a metabolic classification compose less accessory content, and the genes encoding metabolic functions create the most closed pangenome curve. This is in agreement with the high degree of false-positive predictions made by our 35 strain-specific models as GEMs are predictors of what is feasible based on presence of encoding genes but lack regulatory context for expression of those genes. Further, 68.3% of the overall pangenome is classified as uncharacterized, and these gene clusters have the greatest accessory to core ratio and most open pangenome curve, demonstrating the significant knowledge gaps still present for the species. To shed light on uncharacterized genes that may impact the measured metabolic phenotypes, we calculated the biserial correlation between measured phenotypes and presence/absence of gene clusters (Dataset S4). In total, 374 unique gene clusters were found to be positively correlated with one or more phenotypes at a *P* value of <0.001.

### Functional Assessment of the Accessory Genome Provides Discriminatory Power.

We first evaluated the concordance between a single nucleotide polmorphism (SNP)-based phylogenetic tree and one created from a hierarchical clustering of the accessory genome represented in a binary format (*SI Appendix*, Fig. S4). We found that the trees had a correlation of 0.55 and entanglement of 0.12, indicating that accessory genome content is not completely concordant with SNP-based phylogeny. To evaluate the effect of this phenomena on MLST-defined sequence types, we measured the association between accessory genome clusters and defined sequence types (ST) using Cramer’s V statistic (Dataset S5). In total, 9% of accessory gene families were highly associated with more than one ST (361 found in at least two ST with V > 0.4).

Based on this result, we sought to develop an alternative strain typing scheme based on the accessory genome. The *C. difficile* community commonly uses approaches such as SNP trees, PCR ribotyping, and MLST types to distinguish strains. MLST and ribotyping have been shown to be similar in discriminatory capabilities but do not have a direct one-to-one mapping classification of strains (21). A pangenome-based strain typing scheme should resolve groups of strains within a species as well as provide the ability to interrogate the biological relevance of genetic drivers separating different groups. As strain-specific differences have been shown to be critical factors for differentiating phenotypes, such as nutrient niches (41, 51), virulence (52–55), and antimicrobial resistance (56, 57), the ability to distinguish isolates from each other in a way that immediately assigns functional relevance will enhance global epidemiology. To this end, we introduce STAG, an algorithm that capitalizes on the opportunity to classify strain groupings based on the diversity of the accessory gene portfolio.

The STAG algorithm utilizes accessory gene clusters to represent each genome as a binary profile that is defined by gene presence/absence within each accessory gene cluster (*SI Appendix*, Fig. S5). STAG then uses the Jaccard similarity index, defined as the size of the intersection between two binary sets divided by the size of their union, to evaluate how similar each strain vector is to the other (58). Following the calculation of Jaccard similarity, STAG establishes a symmetric matrix composed of pairwise strain similarity, which is used to sort strains into groupings (*Methods*). Next, STAG incorporates the simple metric of compression factor to prioritize strain groupings, which we define as the number of strains divided by the number of groups. Briefly, STAG sorts strains into pangenome types (PGTs) by iteratively passing over the similarity matrix checking for exclusive groupings based on a given threshold of similarity. At each pass, the matrix is sorted according to a range of thresholds, and the threshold that maximizes the compression factor of exclusive groups is selected for that pass. STAG removes the strains of exclusive groups as PGTs, and the threshold identified is set as the new threshold range for the next pass (*SI Appendix*, Fig. S6). For example, in our dataset, a similarity threshold of 0.85 resulted in one exclusive group (21 strains) among the 451 strains, and we deemed this PGT1. The next two iterative sorts identified a similarity threshold of 0.86, resulting in one exclusive group PGT2 (6 strains), and 0.92, resulting in two exclusive groups, which resulted in PGT3 (3 strains) and PGT4 (12 strains), respectively.

**Fig. 4.**
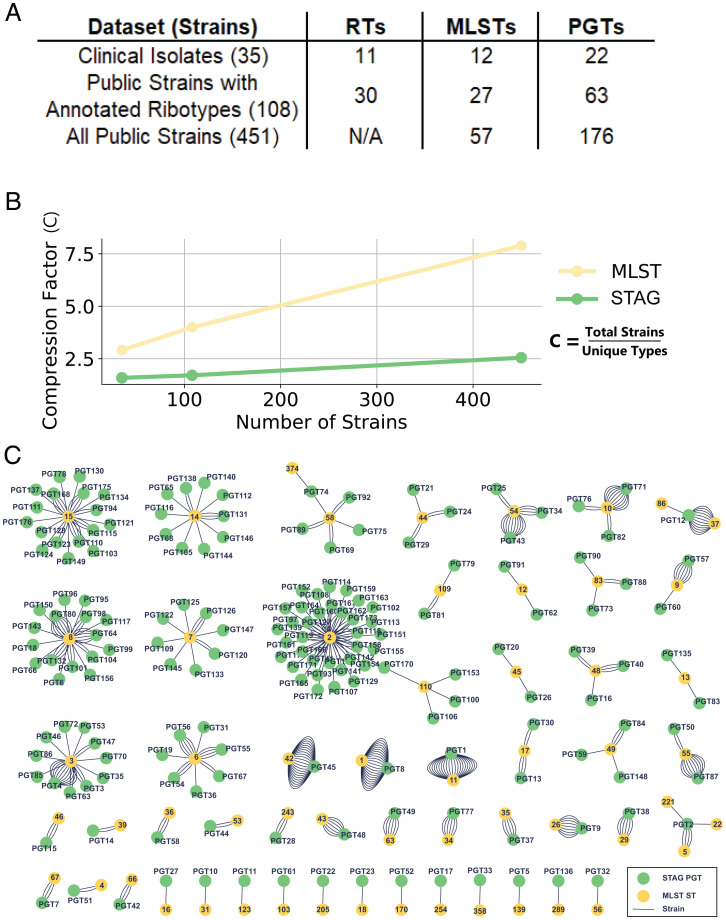
Dataset described via ribotyping, MLST, and STAG and the relative effect of the dataset scale. (*A*) Table describing the three levels within our dataset. Our beginning set of clinical isolates has been ribotyped, and an additional 73 public strains also had ribotyping data. MLST sequence types and STAG PGTs are able to be assigned for all strains. (*B*) The compression factor as a function of the number of strains typed demonstrates that as more strains are considered, strain typing schemes, like MLST, do not maintain their resolution, whereas the STAG scheme is comparatively invariant to the scale of strains considered. (*C*) For each strain, the two types assigned through either STAG or MLST are represented through this network, where the links are each of the 451 strains studied connected to nodes of strain types for each scheme. This analysis highlights the relative number of strains of each type within the dataset as well as the certain MLST types where there is sufficient accessory gene diversity among the strains that STAG establishes numerous different PGTs.

The STAG algorithm categorized our dataset of 451 *C. difficile* strains containing 4,057 accessory gene clusters into 176 PGTs that comprise strain groupings ranging from 1 to 23 strains. We assigned MLST types to each genome using PubMLST (59) (Fig. 4*A*), resulting in a total of 57 STs. Ribotype information was only available for a total of 108 strains in our dataset, limiting our direct PGT–MLST–RT comparisons to 108 genomes (*SI Appendix*, Fig. S7). Given the similar level of discriminatory power between MLST and RT and the paucity of RT data for the public dataset, we used MLST as a baseline to compare strain grouping as a function of the number of strains evaluated (Fig. 4). As the number of strains considered continues to increase, the resolution capabilities of MLST and STAG begin to diverge (Fig. 4*A*). There is an intrinsic trade-off for any strain typing scheme in terms of resolution and compression; each scheme seeks to group strains as efficiently as possible (compression), but these groups must maintain meaning and distinguish strains at scale (resolution). The MLST and RT systems will result in a larger number of strains classified into fewer groups, whereas the PGT maintains flexibility to establish new groups as more genetic content is considered, with each additional strain used to construct the pangenome, and performs similarly in terms of compression factor to cgMLST and SNP-based typing (*SI Appendix*, Fig. S8).

### STAG Types Exhibit an Enhanced Ability to Explain Unique Metabolic Profiles.

We cross-referenced our STAG PGT schemes against the Biolog Phenotype Microarray profiles from the 35 isolates in our experimental dataset to determine the ability of strain type to inform metabolic profile. For these 35 isolates, 28 compounds exhibited differential binary growth capabilities, providing a distinct binary growth vector defining the metabolic profile for each strain (*SI Appendix*, Fig. S1). The distribution of binary growth capabilities across the 35 strains resulted in 26 unique metabolic profiles, where the profile shared by the greatest number of strains (3) was defined by growth-supporting carbon utilization on 3 of the 28 discriminatory compounds by the strains CDH718, CDS009, and CDS079. In turn, the three strain typing schemes classified the 35 strains into 11 distinct PCR ribotypes, 12 MLST sequence types, and 22 STAG PGTs. To study the relationship between these categorical variables (metabolic profiles and strain types), we employed an asymmetric (nonlinear) measure of association by calculating the uncertainty coefficient based on conditional entropy (*Methods*). The uncertainty coefficient indicates what fraction of information can be predicted from one variable when given the other variable. In this case, we are strictly interested in evaluating how well strain type informs the experimental metabolic profile, where a value of zero would be no association and one would be an exact prediction. MLST and RT had uncertainty coefficients of 0.57 and 0.53, respectively, whereas PGT resulted in an uncertainty coefficient of 0.80.

However, this increase is likely a result of the difference in the number of labels to describe the strains by each typing scheme. When using PGTs, the 35 strains are described by 22 labels, whereas the RT and MLST describe the strains as 11 and 12 labels, respectively. We utilized our 35 strain-specific GEMs to generate draft GEMs for all 415 public strains (https://figshare.com/articles/dataset/Dataset9_415_DraftModels_zip/19319906) within our dataset and generated in silico growth predictions as an approximation for metabolic profiles in an effort to evaluate this property at greater scale. The 451 models resolved into 19 in silico metabolic profiles, for which the uncertainty coefficient of the strain typing schemes for MLST and PGT was calculated. Here, the MLST and PGT calculated uncertainty coefficients of 0.85 and 0.92, respectively, as a result in the shift of the relative number of categorical variables at the larger sample size. Overall, in terms of the uncertainty coefficient, the PGT scheme performs more comparably at dataset sizes of 35 and 451 strains when evaluated on the ability to inform on similarly sized sets of categorical variables.

In addition to providing metrics that evaluate a typing schemes’ ability to inform on overall metabolic profiles, examining specific metabolic capabilities illustrates the ability to interrogate functional diversity through STAG PGTs. The niche capability of RT078/ST11 strains to grow using trehalose as a carbon source has recently been associated with virulence implications in CDI (53). The molecular basis for trehalose utilization in RT078/ST11 strains has been attributed to a four-gene insertion, which includes lower-homology second copies of the canonical phosphotrehalase (TreA2) and repressor (TreR2) as well as genes encoding a potential trehalose-specific PTS component (PtsT) and putative glycan debranching enzyme (TreX). We examined the accessory gene clusters used to establish STAG PGTs and identified a total of 12 gene clusters corresponding to this trehalose utilization operon: single-gene clusters for *treX* and *treR2* and five related but distinct gene clusters for both *treA2* and *ptsT* (*SI Appendix*, Fig. S9*A*). The single *treX* cluster along with cluster *treA2_4* and cluster *ptsT_2* are present within 16.1% (73 of 451) of the 451 strains, which include all of the RT078/ST11 strains (21) studied. The single *treR2* cluster along with clusters *ptsT_4*, *ptsT_5*, *treA2_2*, and *treA2_5* are nearly ubiquitous to the overall population, representing 450 of 451, 444 of 451, 445 of 451, 444 of 451, and 391 of 451, respectively. Interestingly, sequences *treA2_3*, *ptsT_3*, and *treA2_1* are uniquely found in strain 1496.1669. Finally, the remaining *ptsT*-related gene cluster (*ptsT_1*) is specific to eight strains classified by STAG as PGT2, wherein the strains here represent a mix of MLST ST5, ST22, and ST221, and critically, this sequence is closest in similarity to the *ptsT_2* cluster including the RT078 strains (*SI Appendix*, Fig. S9*B*). STAG PGTs are based on iterative sequence comparisons as illustrated here, and the resulting PGTs reflect these relationships, allowing for explicit identification of a large number of implicated genetic loci that otherwise would remain undetected.

### PGTs Allow Investigation of Defining Accessory Gene Content.

In addition to providing a means of strain typing that is less subject to a loss of resolution at increasing scale, the PGTs can be interrogated to study functions within the population that drive separation into calculated groups. The 176 distinct PGTs identified among the 451 genomes were compared for gene cluster presence/absence (*Methods*), and defining gene products were examined. These gene clusters are the drivers for inclusion within each PGT and are available in *SI Appendix* and Dataset S5. The annotation information density for each defining group of clusters (presence or absence thereof) was calculated (using the number of genes within a gene cluster with annotation information divided by the total number of genes in the gene cluster and averaged for all clusters identified for a PGT) and used to prioritize gene clusters for deeper study (Table 1). Given the widespread literature on specific ribotype lineages known for being epidemic, we focused on the PGTs that contain the clinically relevant ribotypes RT078, RT027, RT017, RT106, and RT002 in the following sections.

**Table 1.** Pangenome typing containing at least one strain known to be of a hypervirulent ribotype and size of the PGT, number of gene clusters identified, and degree of available annotation information

PGT	Epidemic ribotypes within PGT	PGT size (strains)	No. of presence gene clusters	No. of absence gene clusters	Annotation information density			
					Presence cluster COG	Absence cluster COG	Presence genes	Absence genes
PGT1	RT078	21	124	110	0.298	0.564	0.556	0.854
PGT8	RT027	23	40	27	0.3	0.296	0.55	0.667
PGT12	RT017	13	62	11	0.435	0.545	0.726	0.909
PGT45	RT106	23	6	2	0.167	0	0.167	0
PGT95	RT002	1	10	0	0.2	0	0.3	0
PGT96	RT002	1	20	2	0.3	0	0.1	1
PGT98	RT002	3	12	0	0.083	0	0.167	0
PGT99	RT002	1	7	0	0.143	0	0	0
PGT101	RT002	3	9	0	0.111	0	0.222	0
PGT104	RT002	2	2	0	0	0	0	0
PGT156	RT002	1	12	0	0.167	0	0.083	0

### STAG Reveals That RT078 Strains Contain Unique Zinc Acquisition Genes and Y Gene Previously Implicated in Metal Homeostasis.

**Fig. 5.**
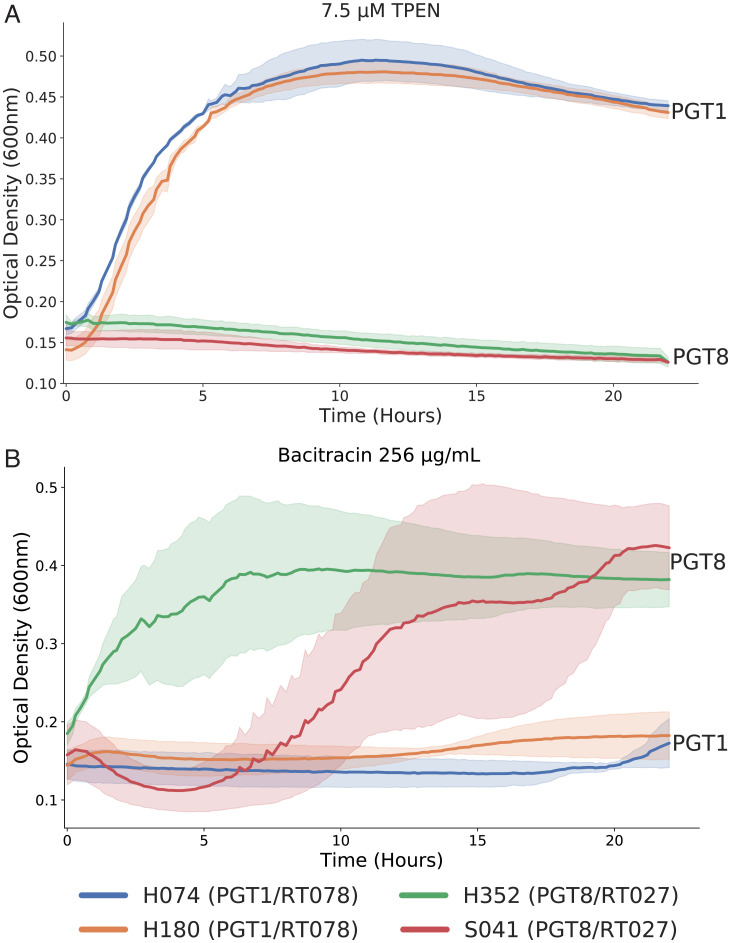
Validation of STAG-predicted phenotypes in PGT1 and PGT8. (*A*) Strains were grown in 7.5 µM TPEN to impose zinc-limited conditions; PGT1 strains H074 and H180 were able to grow in this condition, while PGT8 strains were not (*P* ≤ 0.001, *t* test). (*B*) Strains were grown in 256 µg/mL bacitracin to test resistance capabilities. PGT8 strains H352 and S041 were able to grow in this condition, while PGT1 strains were not (*P* ≤ 0.001, *t* test).

Six clinical isolates from our original dataset are empirically classified as RT078 (CDH074, CDH180, CDH333, CDS009, CDS010, CDS031). These same genomes were classified within PGT1 by the STAG method described here. PGT1 contains a total of 21 strains from the 451 genomes used to define the *C. difficile* PGT scheme, and all strains within PGT1 are also classified by pubMLST as ST11. Given the nature of the sorting algorithm used to construct PGTs, the order in which PGTs arise is an indication of the degree of uniqueness of the group, and this is reflected in the fact that PGT1 is defined by the average presence of 121 gene clusters and the absence of 110 gene clusters in contrast to the population of strains evaluated here. The PGT1 strains represent the most genetically distinct group of the 176 PGTs we have defined, a distinction that aligns with previous studies characterizing the zoonotic prevalence of RT078/ST11 (60–62). STAG PGT classification has identified specific gene clusters that may inform the emergence and virulence of RT078 strains outside of the trehalose utilization discussed above. Specifically, PGT1 contains a cluster annotated as an adaptive-response sensory kinase, *sasA*. In other clinically relevant organisms, *sasA* is responsible for binding to the innate immune receptor glycoprotein DMBT1, promoting bacterial adhesion to tissue within the oral cavity (63, 64). DMBT1 is also found in other tissues, like the lung and small intestine. The presence of *sasA*-positive *C. difficile* strains could provide PGT1 strains an adhesion and colonization advantage over other *C. difficile* strains. A second PGT1-specific gene cluster of interest is the sensor histidine kinase *prrB*. Previous studies indicate that *prrB* is involved in regulating anaerobic metabolism (65, 66). Furthermore, PGT1 includes three additional gene clusters involved in the acquisition and homeostasis of zinc: *znuA*, *znuB*, and *yeiR*. Characterization of the *znuA/znuB* system in *Acinetobacter baumannii* has demonstrated roles in resistance to calprotectin-mediated chelation of zinc, which has been suggested to be a strategy to circumvent nutritional immunity (67, 68). While these genes are present throughout the *A. baumannii* species, these gene clusters are only identified exclusively to PGT1 *C. difficile* strains. The importance of zinc acquisition is further supported by the presence of PGT1-exclusive *yeiR*, which has also been implicated in metal homeostasis in *Escherichia coli* (69). To experimentally validate our predictions for *C. difficile*, we tested PGT1 strains’ ability to grow in zinc-limited conditions by culturing strains in the presence of a zinc chelating agent *N,N,N′,N′*-tetrakis(2-pyridinylmethyl)-1,2-ethanediamine (TPEN) (*Methods*). PGT1 strains, but not PGT8 strains, were able to grow in the presence of 7.5 µg/mL chelator (Fig. 5*A*). Finally, the presence of a tellurium resistance protein TerC is identified as one of the gene clusters driving PGT1 separation. Tellurium resistance genes have been shown to have low levels of divergence, and these resistance genes are thought to be widespread among pathogenic bacteria (70, 71).

### STAG Sheds Light on Endemic, Hospital-Acquired RT027 Strains with Variant Adhesion and Antibiotic Efflux Pump-Encoding Genes.

The most prevalent *C. difficile* ribotype among hospital-associated CDI in the United States is RT027 (72, 73). Strains within RT027 are considered hypervirulent and have persisted as the dominant clone in hospital-associated infections since their emergence in the early 2000s. Our dataset includes two clinical isolates (CDH352, CDS041) from RT027 that are classified by MLST as ST1 and by our pangenome typing method as PGT8. PGT8 also contains an additional 21 publicly available genomes also classified as ST1. PGT8 is defined by 40 present and 27 absent accessory gene clusters, and several of the annotated clusters have potential implications to contribute to the hypervirulent nature of these strains. Like PGT1, PGT8 includes an additional distinct gene cluster annotated as an adaptive-response sensory kinase (*sasA*). With a clustering identity threshold of 80%, we have identified 7 of the 4,057 accessory gene clusters with this annotated function. Each cluster contains genes from a small number of strains ranging from 1.1% (5 of 451) to 6.2% (28 of 451), with certain clusters, such as those identified in regard to PGT8 and PGT1, being exclusive to certain PGTs. The presence of these gene clusters, particularly in groupings that include strains known to be highly problematic, points toward the potential importance of this feature within the evolutionary trajectory of the species. PGT8 contains a gene cluster annotated as *yxdL*, which has been shown to be an ABC transporter participating in a genomic structure of adjacent two-component systems and related ABC transporter, a feature associated with *Bacillus subtilis* and *Clostridia* genomes (74, 75). While the full function of *yxdL* remains unknown, evidence suggesting that it functions as an antibiotic efflux pump is supported by homology to *salX*, which confers salivaricin resistance in *Streptococcus salivarius* (74). Another gene cluster implicated within PGT8 is annotated as *bceB*, a bacitracin export permease protein. Furthermore, the *bce* system is paralogous to the *yxd* system and a component of bacitracin resistance (76). We tested the resistance capabilities of PGT8 strains by culturing in media containing 256 µg/mL bacitracin (*Methods*), and PGT8 stains grew in these conditions, while PGT1 strains did not (Fig. 5*B*). From a metabolic standpoint, PGT8 also contains a gene cluster indicated as *potA*, a spermidine/putrescine transport system that has been studied in *E. coli* (77). Interestingly, spermidine biosynthesis pathway genes and transporter components, including *potA*, have been shown to be up-regulated during temperature and alkali stress in *C. difficile* (78). PGT8 clusters also include the presence of thymidylate synthase and phosphomethylpyrimidine synthase, suggesting isozymes within the species for these functions. Finally, it is worth noting that PGT8 contains IS3 and IS1595 family transposases, indicating potentially consistent mobile elements among the strains.

### STAG Provides Candidate Loci Distinguishing ST37 and ST86 Strains among RT017 Strains.

RT017 is a unique virulent lineage because it is toxinA negative/toxinB positive (79, 80). PGT12 in total contains 13 strains, 3 of which are known to be RT017 (M68, 1141436.4, 1151438.4). All strains within PGT12 are typed by MLST as either MLST37 or MLST86 in agreement with previous studies of this lineage (81). PGT12 is defined by 62 present and 11 absent gene clusters that contain a high degree of annotation information predominated by gene transcriptional regulator annotations. Of note is a cluster annotated as *N*-acetylmuramoyl-l-alanine amidase, which is associated with bacteriophage endolysin activity (82, 83). We also analyzed within PGT12 which gene clusters distinguish the MLST37 from MLST86 strains and were able to identify 75 clusters that contrasted each other within the PGT12, the majority of which remain poorly annotated but do include peptidoglycan acetyltransferase and membrane protein specific to strains of MLST37 and a proline transporter specific to MLST86.

### STAG Highlights Distinct Insertion Sequences within Community-Acquired Strains of RT106.

RT106 reflects the most prevalent community-acquired ribotype according to CDC surveillance and the second most health care–acquired ribotype to date (84). We had three known RT106 strains within our dataset (CDH054, CDH220, CDS057), and all of these strains were grouped into PGT45. There are 23 strains, all MLST42, within PGT45 that are defined by six present clusters and two absent clusters and very limited annotation information overall. Interestingly, PGT45 also contains CDH718, which is known to be RT014 and five of the public strains annotated as RT_SW11. The lone gene cluster with annotation information is annotated as “IS110 family transposase ISFnu3.” This uniquely present mobile genetic element within the strains of a known problematic ribotype could reflect the acquisition of an adaptive trait.

### STAG Demonstrates Divergence in RT002 Strains Despite Convergence of Metabolic Machinery.

The last clinically relevant ribotype of interest was RT002, another highly health care–acquired ribotype, for which there were eight total strains in our dataset (CDS064, CDS065, 1151326.4, 1151354.4, 1151373.4, 1151375.4, 1151403.4, 1151418.4). These RT002 strains, while only classified into ST8 by MLST, were classified into six PGTs: PGT96 (one of eight), PGT98 (two of eight), PGT99 (one of eight), PGT101 (one of eight), PGT104 (one of eight), and PGT156 (one of eight). Although fraught with a paucity of annotation information, we were able to identify notable functional characteristics within this set of PGTs. PGT95 was defined by a cluster annotated as *ydpB*, which is a component of the *ypdA/ypdB* histidine kinase/response regulator pair. Previous studies within *E. coli* demonstrated that this system responds to extracellular pyruvate and is indicated in growth phase–dependent regulation in response to the availability of carbon sources (85, 86). PGT96 was partially defined by two absent clusters that encode penicillinase repressors known to play a key role in the regulation of penicillinase synthesis within gram-positive bacteria (87). The absence of the repressor in this strain could indicate the constitutive expression of the penicillinase synthesis genes and increased antibiotic resistance. Lastly, within PGT156, the gene encoding cell wall–binding protein cwp26 is uniquely present. *C. difficile* is known to produce a number of surface proteins that comprise the S layer; these proteins are suspected to have roles in pathogenesis (88, 89), and the cwp26 contains a putative functional domain of PepSY, which is predicted to have protease inhibition function.

If the pangenome is separated into its constituent functional annotations (Fig. 3 *C* and *D*), the strains can be classified using STAG on specific functional subsections (https://figshare.com/articles/dataset/Dataset_10_Validation_Experiment_zip/19319909). Interestingly, when RT002 strains are typed according to metabolically relevant gene clusters, all strains are grouped into one type of 36 strains (including all 8 RT002 strains in our dataset as well as an additional 28 strains with no ribotype information; all 36 strains are ST8 by MLST). Cluster significance of this metabolically relevant grouping shows that there are seven clusters absent within these strains that are present within 77% of the overall population and another two clusters absent that are present within 58% of the population. Analyzing the functional annotations available for these clusters demonstrates that five of these clusters correspond to various genes within the *yxe* operon, which has been characterized in the related species *B. subtilis* (90–92). The implicated genes within the operon have been shown to be primary transporters of the ABC for polar amino acid uptake and in a more recent study, as key pieces of a disposal route for *S*-(2-succino)cysteine (2SC). 2SC is a product of fumarate-mediated succination of thiols (93), a process implicated in the increase in certain tumors, diabetes, and obesity. The presence of this compound could be used as a biomarker indicating higher levels of cellular aerobic respiration that may result in tumorigenesis, diabetes, and/or obesity (94–96). The absence of this operon within the metabolically clustered RT002 strains may lead to the inability of RT002 strains to use 2SC as a sulfur source, resulting in greater concentrations of 2SC in the gut after invasion of an RT002 strain. Of the remaining absent gene clusters, three are annotated as C4-dicarboxylate transport protein (97), phospho-beta-d-glucosidase *bglH* (98), and l-cystine transport permease protein, and one cluster is annotated with no valuable annotation information. The C4-dicarboxylate transport protein–encoding gene has been shown to be a participant of the sigma G regulon in sporulation and its product detected in *C. difficile* spores.

## Discussion

In this study, we perform a functional analysis of the *C. difficile* pangenome in an effort to increase understanding of strain-specific traits in terms of both genotype and phenotype. Taking a systems biology approach enabled us to identify and contextualize important genetic and phenotypic features within the vast diversity of this species. Motivated by the importance of specific carbohydrate and bile acid metabolism in *C. difficile* pathogenesis (55, 99–101), we metabolically profiled 35 clinical isolates and investigated their diverse capabilities. The wide array of growth dynamics exhibited from our high-throughput screening necessitated sophisticated data analysis, which was facilitated by the use of GP regression models. These two techniques demonstrated through variable growth modalities that catabolic capabilities were diverse at a strain-specific level, including differences across strains of the same PCR ribotype and MLST sequence type. Following the identification of unique carbon source utilization profiles, strain-specific GEMs of metabolism were generated for each isolate to bridge the genotype to observed phenotypic diversity and infer potential mechanistic insight. The in silico simulations recapitulated the majority (76%) of growth phenotypes. However, there were a high number of false-positive error mode predictions, which indicated that the models of metabolism, which are predictors of all theoretically possible growth capabilities based on enzymatic coding gene content, were lacking the biological context concerning transcriptional regulation and/or enzyme efficiency that restrict capabilities in vitro (102).

To robustly explore all the genetic diversity outside of the metabolic network, we constructed the pangenome of *C. difficile* with the inclusion of an additional 416 public genomes. Characterizing the pangenome demonstrated different conservation levels across various functional categories. We developed the STAG algorithm to type our group of 451 strains based on the accessory genome. STAG established meaningful groupings of strains that corresponded to experimentally derived phenotypic differences. The method is distinct from other typing methods that rely solely on the core genome. STAG highlights differences in horizontal evolution, which is a significant factor when studying bacterial species.

Applying STAG to the *C. difficile* pangenome identified genes that strongly contributed to unique groupings of strains based on their contrasting presence and absence from the overall population. STAG highlighted diverse functions ranging from specific transporters, sensory responses, and two-component systems to cell wall proteins across the clusters driving separation of PGTs containing known epidemic lineages. An especially valuable aspect of the approach is identification of a large and diverse number of genetic loci that differentiate strains. These loci present critical candidates for further characterization and improvement of annotation to increase understanding of pathogenesis at the species level. While specific clinical relevance remains unclear, STAG could be valuably applied to strain groups with associated clinical outcomes to identify associated gene clusters.

Overall, the results presented here suggest the importance of a genomics-driven approach to understand *C. difficile* diversity and identification of the evolutionary events leading to propagation of epidemic lineages. Trait acquisition has been demonstrated across functional categories, and most pressing is the vast amount of genetic content that remains uncharacterized. The high percentage (74.5%) of implicated present genes with poor to no annotation information within the gene clusters driving separation of PGTs demonstrates that overall characterization of genes lacking experimental evidence of function (the “y-ome”) (103) for *C. difficile* remains high. Unsurprisingly, these clusters exhibit the highest degree of openness within the subdivisions of the pangenome, and likely, these clusters contain genes that are critical factors in the evolutionary trajectory and history of *C. difficile*. Our exploration of total gene content has suggested that an investigation into the transcriptional regulatory network of *C. difficile* would prove informative. The processes involved and related to regulation appear to be critical in differentiating strains, and an accurate description of the transcriptome in presumed physiological conditions during infection would provide a crucial systems-level explanation of cellular response. Use of machine learning methods on high-quality expression profiles has been shown to provide such a window into understanding transcriptional regulation in *E. coli* and *Staphylococcus aureus* (104, 105) and with proper datasets, could be applied to *C. difficile*.

The insights into the accessory genome and its specific components to groups of strains presented here have added to the overall understanding of *C. difficile* and provided a means for bringing the important factor of genetic diversity to the forefront. The STAG method presented has advantages in maintaining flexibility with the scale of strains studied, reliance solely on WGS data, the ability to identify functional differences across PGTs, and the illumination of genetic loci with discriminatory power. STAG is unique in its untargeted approach that does not require user-defined thresholds, thus making it a straightforward and valuable addition to the suite of pangenome analysis methods. In any strain typing scheme, there will be a trade-off between compression and resolution of the resulting groups in that each scheme strives to establish meaningful groups that capture the relationship among strains. Given the continued growth of genome sequences available for most bacterial species, methods that leverage these data to identify key genetic features in relation to populations will be important to the future of global epidemiology. Future endeavors in characterization in concert with data analytics will enhance the scientific knowledge of the *C. difficile* species commensurate with the promise of omics big data.

## Methods

Methods for phenotypic profiling with biolog microarrays are detailed in *SI Appendix*, text S3. Processing of growth data through GP regression is detailed in *SI Appendix*, text S4. Methods for WGS are detailed in *SI Appendix*, text S5. Methods for constraint-based modeling FBA are detailed in *SI Appendix*, text S6. Methods for strain-specific model creation are detailed in *SI Appendix*, text S7. Methods for pangenome construction and analysis are detailed in *SI Appendix*, text S8. Methods for phylogenomic analysis are detailed in *SI Appendix*, text S9. Methods for calculation of the uncertainty coefficient are detailed in *SI Appendix*, text S10. Methods for Jaccard similarity toward establishing strain groups are detailed in *SI Appendix*, text S11. Methods for identification of gene clusters driving PGT separation are detailed in *SI Appendix*, text S12. Methods for the validation experiments conducted are detailed in *SI Appendix*, text S13.

## Supplementary Material

Supplementary File

Supplementary File

Supplementary File

Supplementary File

## Data Availability

All biolog data, 35 strain genome sequences, 35 strain-specific models, 415 draft strain-specific models, and validation experiment data have been deposited in FigShare (https://figshare.com/articles/dataset/Dataset_6_Biolog_Time_Series_Data/19319897, https://figshare.com/articles/dataset/Dataset_7_Isolate_Genomes_zip/19319903, https://figshare.com/articles/dataset/Dataset8_CuratedStrainSpecificModels_zip/19319900, https://figshare.com/articles/dataset/Dataset9_415_DraftModels_zip/19319906, and https://figshare.com/articles/dataset/Dataset_10_Validation_Experiment_zip/19319909).
