# Supplementary material for: Systems biology approach to functionally assess the Clostridioides difficile pangenome reveals genetic diversity with discriminatory power
Source: Proc Natl Acad Sci U S A. 2022 Apr 27;119(18):e2119396119. doi: 10.1073/pnas.2119396119 (PMC9170149; doi:10.1073/pnas.2119396119)
Supplement: Supplementary File [file pnas.2119396119.sapp.pdf]

Supplementary Information:

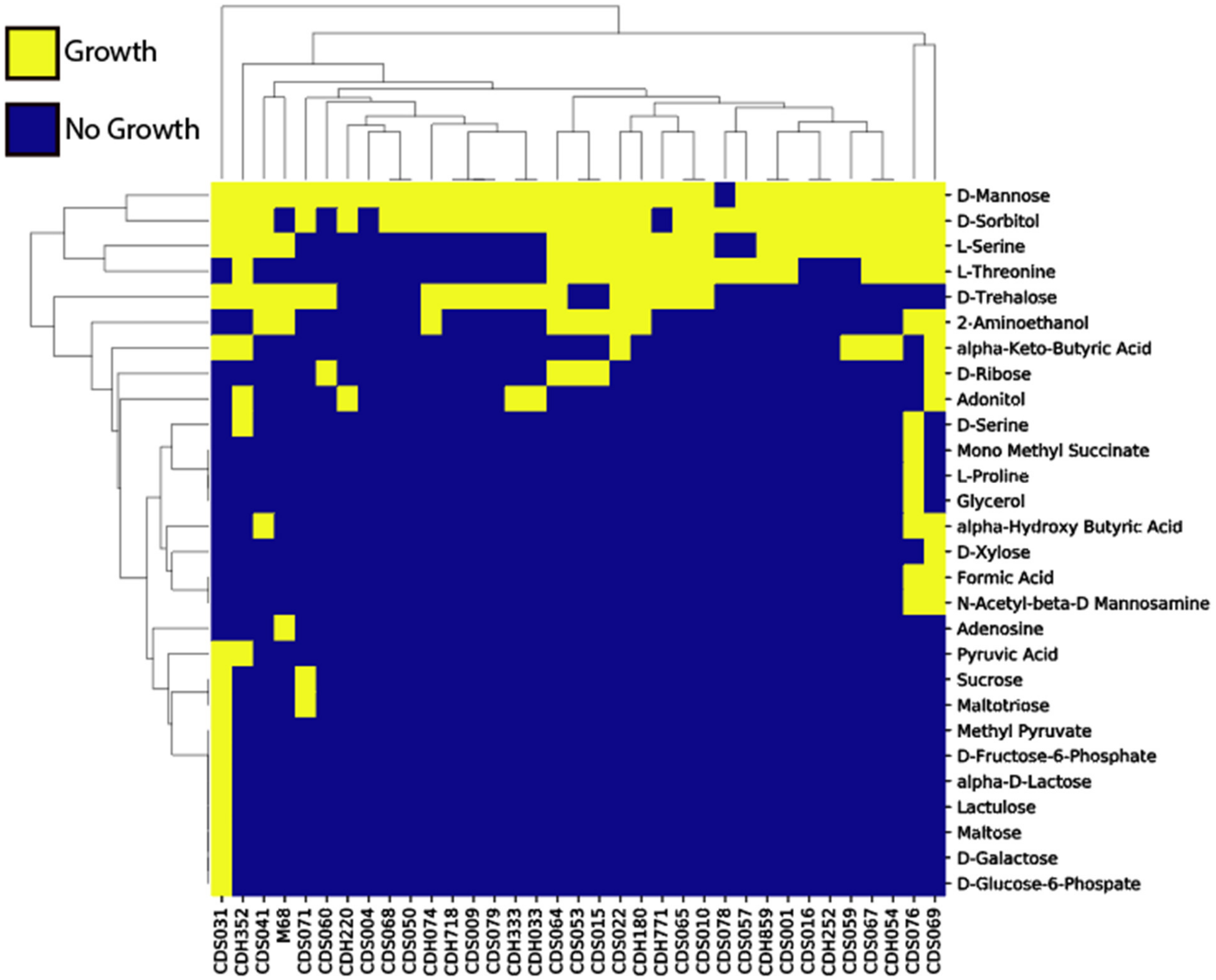

Supplementary Figure 1: 28 Non-Unanimous Growth Supporting Carbon Sources. The carbon sources where strains varied in terms of growth capabilities and as a result define the varying metabolic profiles.

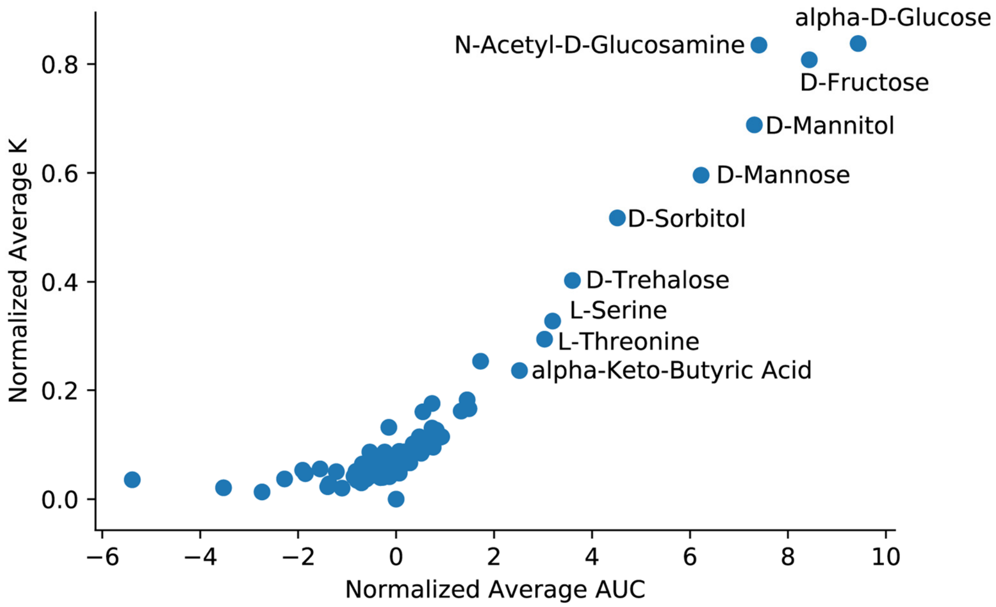

Supplementary Figure 2: Average fit parameters across 35 isolates. After averaging the AUC and K, the most growth supporting nutrients across our dataset emerge.

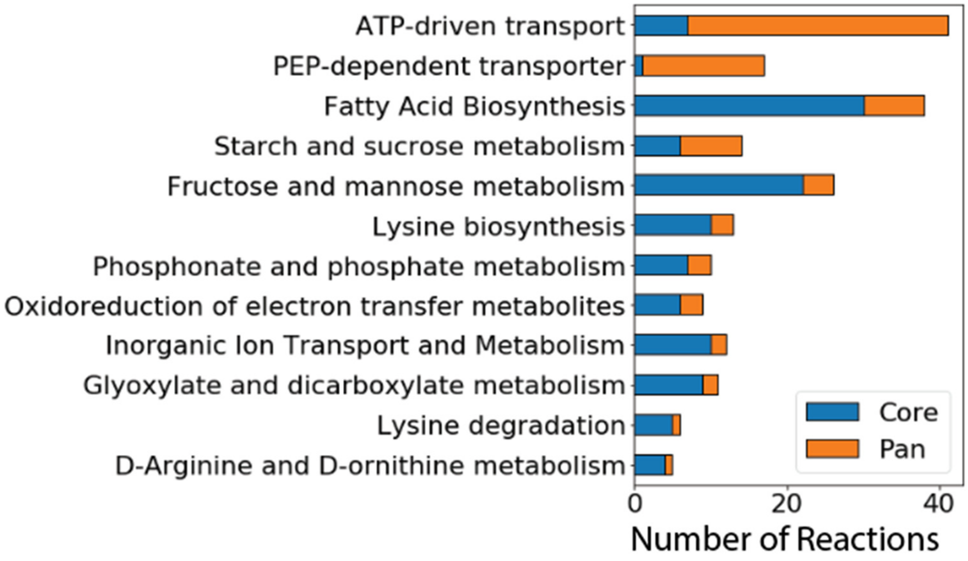

Supplementary Figure 3: **Reaction Subsystems with High Degree of Non Conserved Genes.** Pictured are the subsystems identified through the use of GEMs where greater than 15% of the reactions in the subsystem contain at least one non conserved gene within the set of 35 clinical isolates.

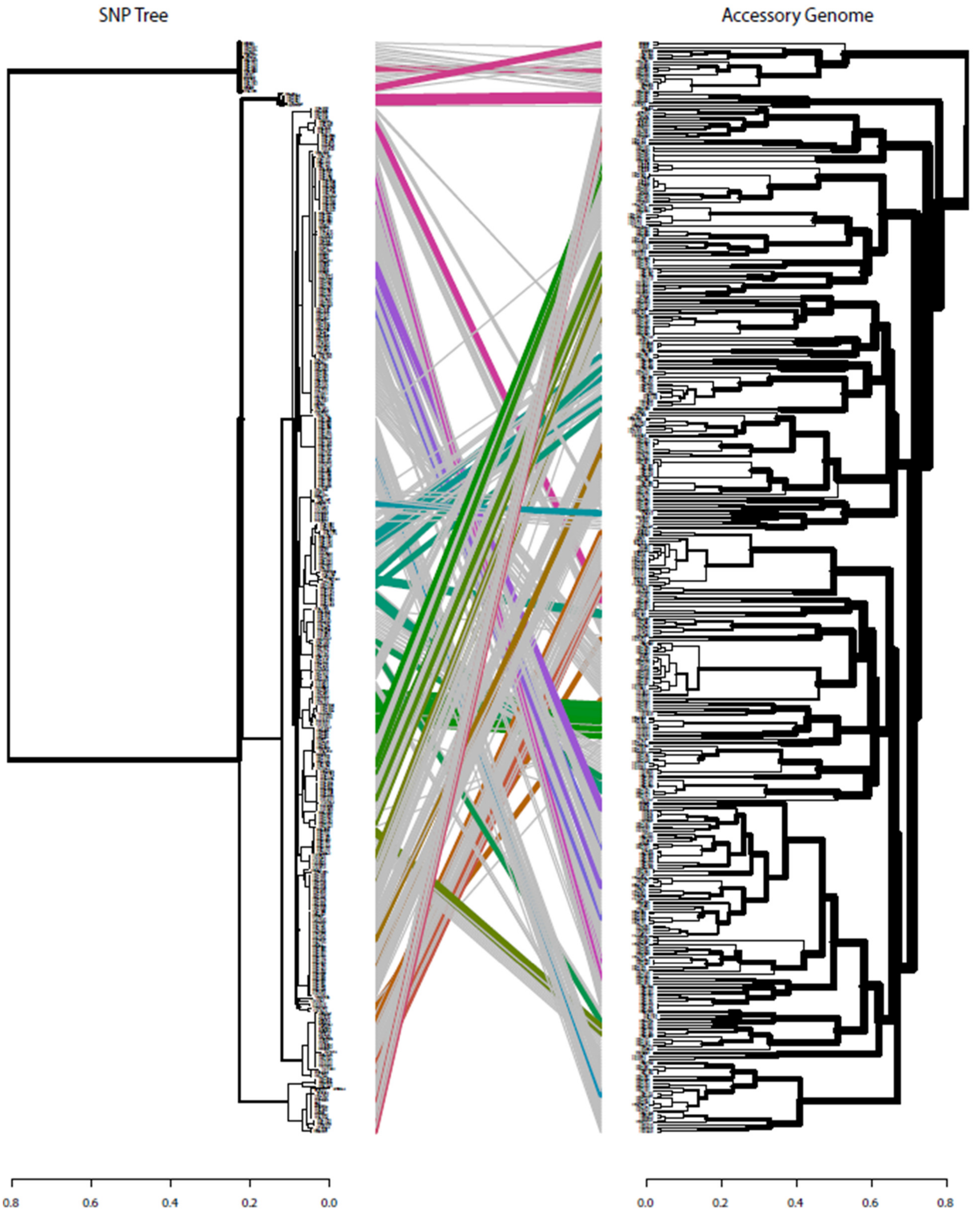

Supplementary Figure 4: Comparison of SNP-based dendrogram (left) and accessory-genome based dendrogram (right). Colored lines connecting the two trees indicate groups of strains with identical clustering hierarchies, gray lines indicate different clustering hierarchies. The two trees

have a correlation of 0.55 and entanglement of 0.12 indicating that accessory genome content is not completely concordant with SNP-based phylogeny. Tree comparisons and visualizations were constructed using the dendextend package (see **Methods**).

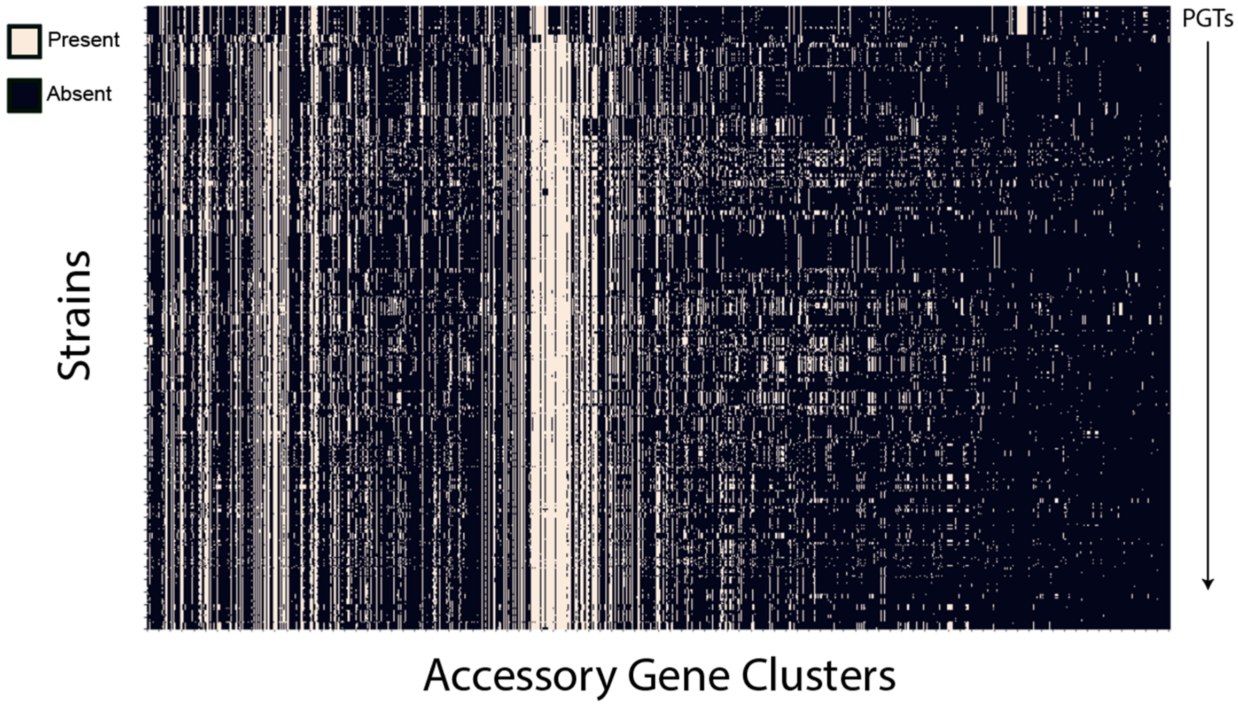

Supplementary Figure 5: Accessory gene cluster presence absence for each strain. Pictured is each of the 451 strains (ordered by eventual PGT) and presence/absence of each of the 4,057 accessory gene clusters.

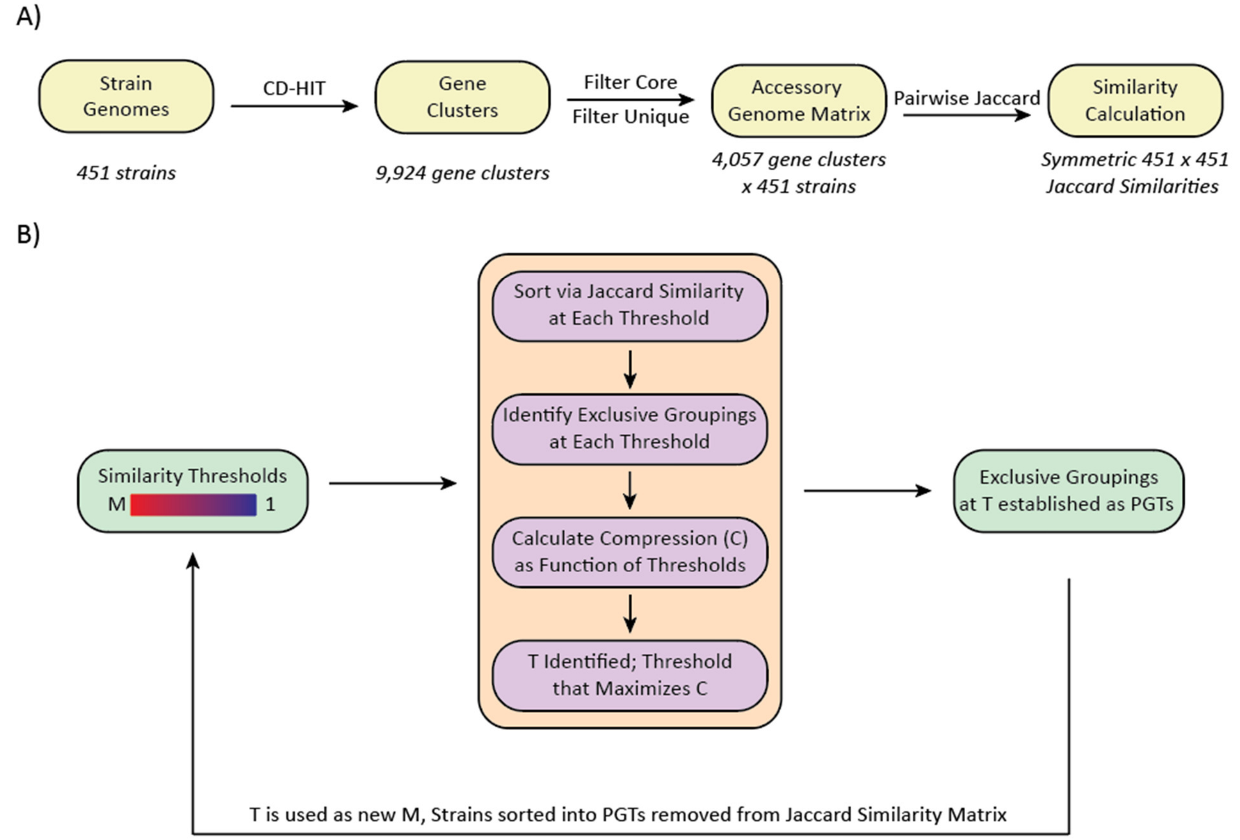

Supplementary Figure 6: **STAG Workflow for Establishing Pan-Genome Typings. A)** Strain genomes are first clustered using panX to establish the accessory genome. The Jaccard Similarity was calculated on accessory genome vectors between strains. **B)** A range of similarity thresholds are evaluated at each iterative pass of the sorting algorithm over the jaccard similarity matrix. Exclusive groupings are identified at each potential threshold and the threshold that maximized compression of exclusive groups is selected. Exclusive groups become PGTs and the identified threshold is the beginning threshold range, M, of the next pass. The strains sorted into an exclusive PGT are dropped from the similarity matrix.

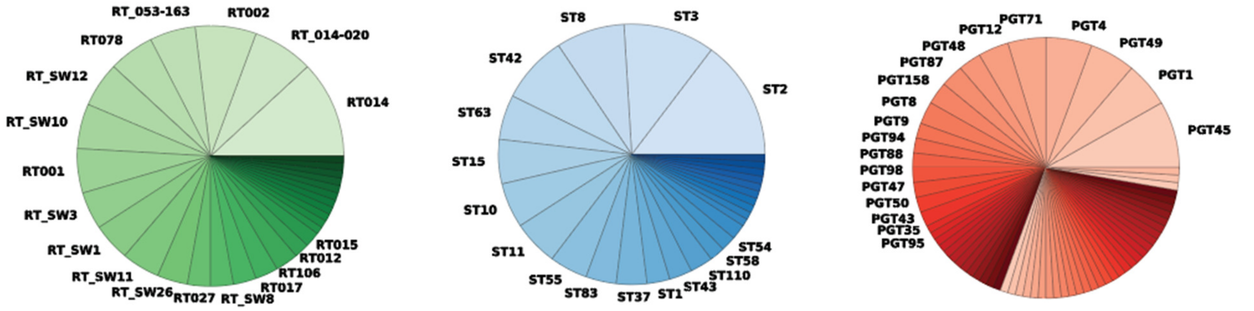

Supplementary Figure 7: **Assigned Typings for All Strains with Ribotyping Information.** For the 108 strains for which a typing is available in all three schemes, the relative composition

of typings is shown. While there are strains of unique RT and MLST, there are a far greater number of strains that are assigned as unique PGT.

A

Dataset (Strains)	RTs	MLSTs	PGTs	CGMLST	KMER	SNP	Hierarchical
Clinical Isolates (35)	11	12	22	16	32	17	14
Public Strains with Annotated Ribotypes (108)	30	27	63	40	98	60	36
All Public Strains (451)	N/A	57	176	172	312	146	99

B

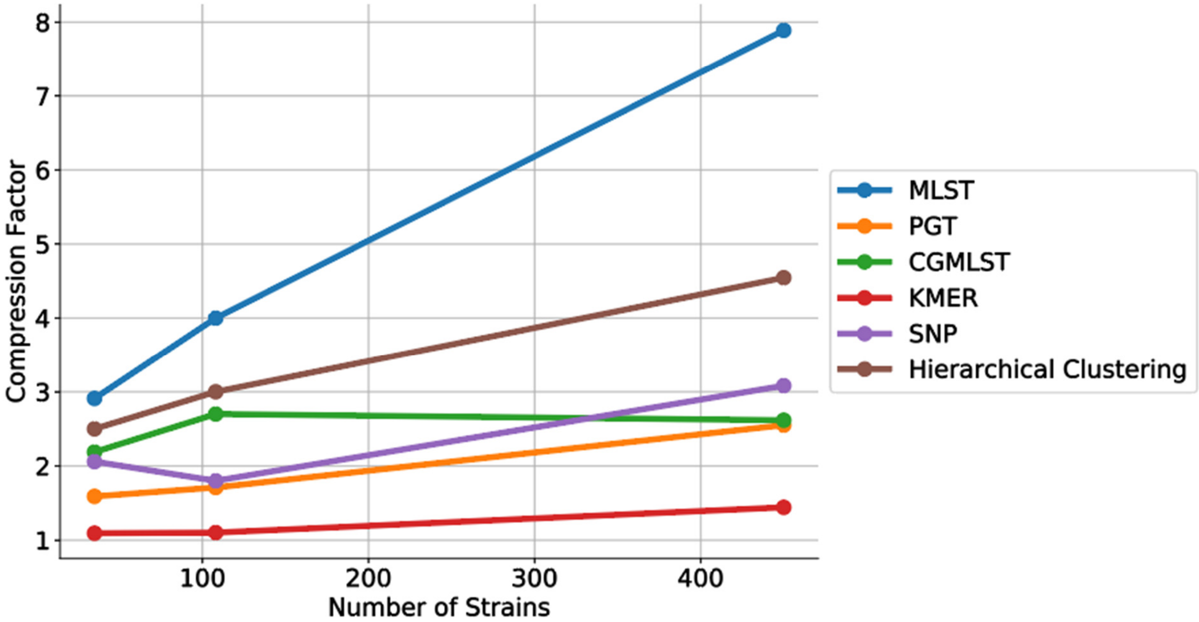

Supplementary Figure 8: Expanded comparison of different strain typing schemes. A) Number of strain groups identified as detailed within Figure 4 expanded to include CGMLST, KMER, SNP, and hierarchical clustering of the accessory genome. **B)** The compression factor as a function as a function of the number of strains typed demonstrates that the use of a single distance metric in hierarchical clustering results in groups in between MLST and CGMLST, SNP, and STAG in terms of resolution.

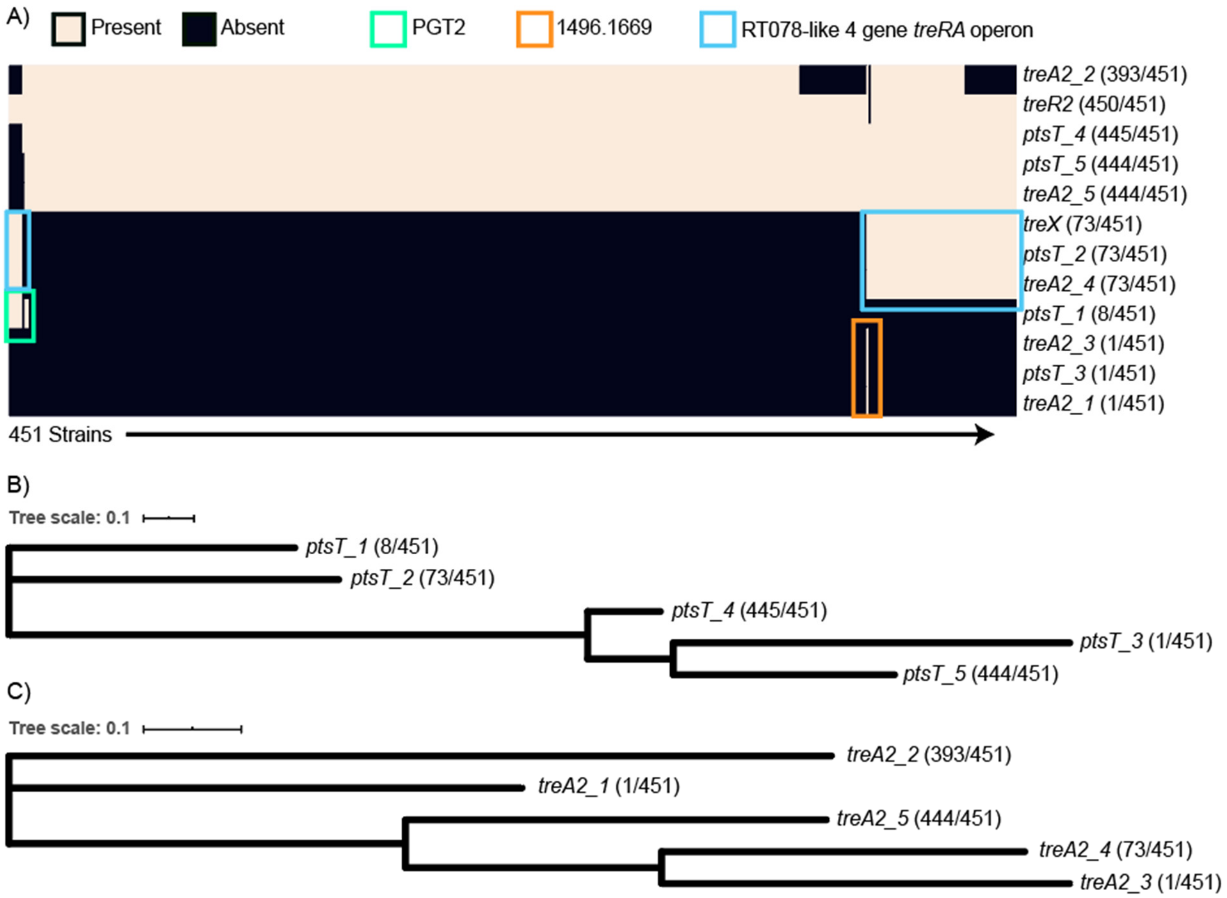

Supplementary Figure 9: **Analysis of *treRA* operon characteristic to RT078 and related gene clusters. A)** We identified the gene clusters within the pan-genome that include the sequences of a previously identified four-gene insertion responsible for increased ability to catabolize trehalose by RT078 strains. These clusters are present within 73 strains studied, including all strains of PGT1 which includes all RT078 strains within the study. The remainder of the strains represent a variety of Clade 1 strains that have also been shown to potentially include this gene insertion. By analyzing the identified related clusters to the *treA2* and *ptsT* gene clusters of the insertion we were able to identify variants unique to one strain as well as a variant *ptsT* specific to strains classified as PGT2. **B)** The alignment of the representative sequences for *ptsT* clusters demonstrates that the PGT2 specific variant is of closest relation to the known *ptsT* indicative of RT078. **C)** The alignment of the representative sequences for *treA2* clusters demonstrates that of the two unique variants to strain 1496.1669 one is more related to that within the RT078 operon and the other to genes more core genes in the population.

Supplementary Datasets:

Dataset 1: Fold Change, Max OD, and Fit Parameters for Each Strain on all Substrates
Dataset 2: All Public Genomes used to construct the pan-genome
Dataset 3: PGTs and Associated Cluster Information

Dataset 4: Pan-genome matrix and association to observed phenotypes
Dataset 5: Accessory Genome and Sequence type associations
Dataset 6: All Biolog Data (https://doi.org/10.6084/m9.figshare.19319897)
Dataset 7: 35 Strain Genome Sequences (https://doi.org/10.6084/m9.figshare.19319903)
Dataset 8: 35 Strain-Specific Models (https://doi.org/10.6084/m9.figshare.19319900)
Dataset 9: Draft Strain-Specific Models (https://doi.org/10.6084/m9.figshare.19319906)
Dataset 10: Validation Experiment Data (https://doi.org/10.6084/m9.figshare.19319909)

Supplementary Text:

S1. Functional Annotation Specific Driven Typing

In addition to using the full accessory genome to define strain groupings, we were interested in evaluating each functional annotation subset ability to drive typings. The full set of accessory genes was split into the 4 categories of Metabolism, Information Storage and Processing, Cellular Processes and Signaling, and Uncharacterized as described within the section "Characterizing the *C. difficile* pan-genome". Each of these 4 sets were then independently used following the same algorithm described when analyzing the full accessory genome. When using these subsets the 451 strains can be grouped into 94, 166, 182, and 209 groups based on accessory metabolic, information related, signaling related, and uncharacterized genes respectively. Grouping based on these subsets allows for insight into the differential similarity as exhibited by the RT002 strains which did not group into a singular all accessory content, but were exclusively grouped by only the metabolic related accessory genes.

S2. Expanded Comparison to Additional Typing Schemes

In addition to our presented comparisons to the most-widely used MLST and PCR Ribotyping schemes, we also compared STAG to other available typing schemes that consider increased genetic content. We were able to establish groups through SNP-based, KMER-based, and a core-genome MLST (CGMLST) approach. Additionally, we also analyzed the ability of standard distance-based clustering methods, namely hierarchical clustering, to derive strain groups on the same accessory genome matrix used as input to STAG. These comparisons serve to evaluate two key aspects of the STAG approach: 1) the effect of increased amount of genetic content used to establish groups, and 2) the iterative sorting based on shifting similarity thresholds to establish groups versus an existing clustering technique.

Overall, the SNP and CGMLST strain types had similar compression to the STAG PGTs, however a slightly greater percentage (59.6%, 60.4% respectively versus 55.6%) of the final strain types in each case were single strain sized (Supplementary Figure 8). The KMER approach failed to establish meaningful groups and only compressed the 451 strains into 312 groups. Conversely, the hierarchical clustering based groupings resulted in 99 strain groups compared to the 176 PGTs with 48.5% of these groups being single strain groups. On average each typing scheme of STAG, CGMLST, KMER, and SNP resulted in comparable average strain group sizes of 2.56, 2.62, 1.44, 3.09 respectively. The use of hierarchical clustering necessitated the specification of a single distance threshold on which to define groups (.05 here) and the lack of flexibility in distance metrics leads to larger groups encapsulating more diverse strains more similarly to the MLST system. The largest group of strains when using

hierarchical clustering is 68 strains, which results in limited interpretability. The goal of any strain-typing scheme is to provide a means to describe the variation of strains into meaningful groups. To this end each approach has its strengths and careful consideration of what content drives the resulting strain types is important. STAG presents another valuable tool that is easy and efficient to use and offers a high degree of interpretability and information in regard to accessory genome content.

S3. Phenotypic Profiling by Biolog

Strains were cultured in BHI medium (Difco) supplemented with 0.5% (w/v) yeast extract (Fisher Scientific) overnight (~16 hours) in an anaerobic chamber (5% hydrogen, 90% nitrogen, 5% carbon dioxide). 1 ml of overnight culture was diluted into 10 ml of defined minimal media with previously described composition (106) and 100 µl was added to each well of Biolog Phenotypic Microarray plates (PM1 and PM2). Growth assays were performed under anaerobic conditions with optical density at 620 nm read every 10 minutes following 5 seconds of shaking over a period of 16 hours.

S4. Gaussian Process Regression Models of Growth

Correlation between experimental replicates were evaluated and replicates passing this quality check (Pearson R>0.7) were pooled for the following analysis. One of the advantages to using gaussian process (GP) regression is the ability to pool biological replicates and makes this approach particularly suited to high-throughput screens. GP to infer microbial growth parameters was conducted using the AMiGA (107) program through the default settings presented by AMiGA. For each strain the pooled quality replicates were log transformed and negative control subtracted at each time point. A GP regression model was then fit for each pooled time course and growth parameters inferred resulting in identification of the growth parameters for each strain on each of the 95 carbon sources.

The carrying capacity (K) value represents the maximum population size and the area under the curve (AUC) value is a measurement of net growth over time irrespective of curve shape, therefore these metrics are particularly suited for high throughput screens of divergent strains exhibiting varied growth dynamics. Potential thresholds for each growth parameter (AUC or K) were evaluated on the range from minimum to maximum parameter value across all strains. Sensitivity analysis for each parameter was executed by binarizing experimental data using each potential threshold, while holding the complementary parameter threshold constant. This led to identification of greater than 1.25 AUC and greater than .3 for K to jointly define growth calls of the experimental data. Implementation of a combined threshold increases confidence of growth analysis by minimizing impact of data noise and variant growth dynamics as compared to simple fold change or maximum optical density threshold growth calling (108). This method serves to minimize the impact of incidental spikes and fluctuations, which can lead to faulty growth calls when using just fold change or optical density to binarize high-throughput data.

S5. Whole Genome Sequencing

Cryofrozen isolates of each *C. difficile* strain were incubated on Brain Heart Infusion (BHI) agar under anaerobic conditions for 24-48 h. Genomic DNA was extracted using the MasterPure Complete DNA & RNA Purification kit (Lucigen, MC85200) and libraries of fragmented genomic DNA were prepared using NEXTflex Rapid DNA-Seq Kit (Bioo Scientific, NOVA-5149-02). Paired-end reads (2 x 150 bp reads) were generated on the MiSeq platform (Illumina, San Diego, CA, USA) using the Illumina MiSeq Reagent Kit v2 (MS-102-2002) and PhiX Control Kit v3 (FC-110-3001). WGS reads have been submitted to NCBI as BioProject PRJNA472399.

S6. Constraint-based modeling flux balance analysis

Constraints-based analyses were conducted using the COBRApy toolbox. For the *in silico* growth simulation of sole carbon source utilization the minimal media (109) was used and glucose was removed and all other carbon source exchange reactions were opened in an iterative fashion to evaluate if growth was possible (31, 36, 43, 48, 50). Growth versus no growth was determined through flux balance analysis in each condition, optimizing for the biomass function. Within these simulations we consider biomass objective flux of greater than zero designated carbon sources that supported growth.

S7. Strain-Specific Model Creation

A standard strain-specific model generation protocol (47) was followed to generate draft strain-specific models for the dataset of 35 isolates as well as 415 publicly available strains. In the case of the 35 isolates the outputs of these 35 isolates were further curated based on false negative predictions identified through comparison to our experimental dataset (110). This manual curation involved identification of reactions and genes through literature curation as well as homology with other related species. Following the curation of the 35 strain-specific models, the isolate closest in terms of phylogeny to each public strain was used as the base model for generation of draft models for the public strains.

S8. Pan-Genome Construction and Analyses

A total of 1,246 whole-genome sequences of *C. difficile* were downloaded from the PATRIC database (111) on August 25, 2019. To filter for high-quality genomes a cutoff of assemblies composed of 100 or fewer contigs was applied. Furthermore, an MLST analysis of the genomes was performed using MLST (60). All genomes that could not be assigned to an MLST type or species were also filtered out. This led to a final set of 415 genome sequences for downstream analysis. Sequence homology was used to cluster genes into gene families using panX version 1.5.1 (112). These gene families were then used to designate core and pan genes by identifying the gene families found in less than 1% of the total 451 strains. Biserial correlations between all gene clusters and measured phenotypes were calculated using the pointbiserial function in the scipy stats package. The Cramer's V statistic for categorical-to-categorical association was used to evaluate associations between pan-genome clusters and ST types. This calculation was implemented in python and uses the correction from Bergsma and Wicher (113).

S9. Phylogenomic Analysis

Phylogenomic analysis was performed using GToTree (115) on the clinical isolate dataset along with 415 public strains. Prokka FASTA files were used as inputs to GToTree analysis and the resulting tree was visualized using the Interactive Tree of Life web-based tool (115, 116). This tree was compared with a dendrogram constructed using a binary representation of the accessory genome (451x4,057 maxtrix where 1=gene cluster present, 0=gene cluster absent). The dendrogram was constructed using jaccard distances and the complete clustering method. The two trees were compared (including calculation of correlation and entanglement) using the dendextend package in R (117). Clustering methods were compared to those of cgMLST (extracted from enterobase(29)) and KMERs obtained using the SKA package (118).

S10. Calculation of Uncertainty Coefficient

The uncertainty coefficient is calculated by definition where the two discrete variables are given as each strain typing classifications, T and metabolic profile, M. The conditional distribution is defined through Equation 1 and the entropy of the single distribution as Equation 2. Conditional

entropy follows as Equation 3 and lastly the uncertainty coefficient is calculated as defined by Equation 4.

$$(1)\quad P_{M|T}(m,t) = P_{M,T}(m,t)/P_T(t)$$

$$(2)\quad H(M) = -\sum_m P_M(m) log P_M(m)$$

$$(3)\quad H(M|T) = -\sum_{m,t} P_{M,T}(m,t) log P_{M|T}(m|t)$$

$$(4)\quad U(M|T) = \frac{H(M)-H(M|T)}{H(M)}$$

S11. Using Jaccard Similarity to Establish Strain Groups

Using the constructed pan-genome the accessory gene clusters (those that are not core nor unique) were identified. These 4,057 gene clusters were used to define a vector of presence/absence for each strain in terms of whether or not that strain had genes present within the gene cluster. The Jaccard Similarity coefficient was calculated between each of these 451 binary vectors of length 4,057 and used to establish a symmetric 451x451 matrix of the similarity coefficients. An iterative sorting workflow was developed that parses through this matrix and establishes pan-genome typings. A range of potential similarity thresholds is established and the set of strains meeting the current Jaccard similarity matrix threshold for each single strain are identified. From this information exclusive groups (i.e. at the threshold, every strain similar to the current strain is also sufficiently similar to every other strain similar to a given strain) are identified on a per threshold basis. Out of these exclusive groups the compression factor (Number of Strains in Exclusive Groups/ Number of Exclusive Groups) is calculated and the threshold maximizing this compression factor is selected for this iteration over the Jaccard Similarity matrix. The exclusive groups are established as PGTs and corresponding strains are filtered from the Jaccard Similarity Matrix for the next sorting pass. Additionally, the compression maximizing threshold is the starting point of similarity threshold range. This process is repeated until the similarity threshold has been incremented to only identify single strains into PGTs.

S12. Identification of Gene Clusters Driving PGT Separation

For comparison of each PGT the binary presence/absence vectors of each strain within the group were averaged to calculate the representative gene cluster portfolio for each PGT. The PGT-specific mean and all strain population mean were compared to identify divergent clusters. We consider a gene cluster present in less than 20% of the population to be predominantly absent in the overall population and a gene cluster present in greater than 90% of the population to be predominantly present in the overall population. The absolute value of the difference between mean presence in population versus mean presence in a specific PGT was calculated for each cluster and used to designate as either predominantly present or absent. In each case if the absolute value of difference was greater than 0.95 the cluster was identified as being present or absent the given PGT in contrast to the population. For example if a gene cluster was determined to only be present within 15% of the overall strains, but was present in 100% of the strains of a given PGT, this cluster would be identified as a driving cluster in terms of gene presence for that PGT. For each cluster identified in this way the functional annotations at both the gene cluster level (in terms of COG) and gene annotation level (in terms of function) were evaluated. The information density (ratio of clusters with annotation information to total clusters) was calculated for each PGTs defining clusters in terms of both presence and absence. To ensure that information was not simply more available for PGTs with a greater number of clusters identified the size of PGT in terms of number of strains and number of clusters driving separation of the

PGT was evaluated (Number of Presence Clusters R=.145 , Number of Absent Clusters R=.372). Additionally, correlation between PGT size and information density was evaluated (Number of Presence Clusters R=.249 , Number of Absent Clusters R=.089).

S13. Validation Experiments of STAG Predicted Traits

Strains were cultured overnight as for Biolog profiling, except that following overnight incubation cells were diluted into defined medium at an OD value of 0.05 and incubated until mid-log growth was achieved. Exponentially growing cells were then back diluted to a starting OD of 0.1 in 200 µl of defined medium supplemented with Bacitracin (Sigma Aldrich) or N,N,N',N'- tetrakis-(2-Pyridylmethyl)ethylenediamine (TPEN) (Sigma Aldrich) as indicated.

SI References

106. J. R. Fletcher, S. Erwin, C. Lanzas, C. M. Theriot, Shifts in the Gut Metabolome and Clostridium difficile Transcriptome throughout Colonization and Infection in a Mouse Model. *mSphere* **3** (2018).
107. F. S. Midani, J. Collins, R. A. Britton, AMiGA: software for automated Analysis of Microbial Growth Assays. mSystems. **6**, (2021)
108. C. J. Norsigian, E. Kavvas, Y. Seif, B. O. Palsson, J. M. Monk, iCN718, an Updated and Improved Genome-Scale Metabolic Network Reconstruction of Acinetobacter baumannii AYE. *Front. Genet.* **9**, 121 (2018).
109. T. Karasawa, S. Ikoma, K. Yamakawa, S. Nakamura, A defined growth medium for Clostridium difficile. *Microbiology* **141 (Pt 2)**, 371–375 (1995).
110. I. Thiele, B. Ø. Palsson, A protocol for generating a high-quality genome-scale metabolic reconstruction. *Nat. Protoc.* **5**, 93–121 (2010).
111. A. R. Wattam, *et al.*, PATRIC, the bacterial bioinformatics database and analysis resource. *Nucleic Acids Res.* **42**, D581–91 (2014).
112. Wei Ding, Franz Baumdicker, Richard A Neher, panX: pan-genome analysis and exploration, *Nucleic Acids Research*, Volume 46, Issue 1, 9 January (2018).
113. W. Bergsma, A bias-correction for Cramér's V and Tschuprow's T. *J. Korean Stat. Soc.* **42**, 323–328 (2013).
114. M. D. Lee, GToTree: a user-friendly workflow for phylogenomics. *Bioinformatics* **35**, 4162–4164 (2019).
115. I. Letunic, P. Bork, Interactive Tree Of Life (iTOL) v4: recent updates and new developments. *Nucleic Acids Res.* **47**, W256–W259 (2019).
116. I. Letunic, P. Bork, Interactive tree of life (iTOL) v3: an online tool for the display and annotation of phylogenetic and other trees. *Nucleic Acids Res.* **44**, W242–5 (2016).
117. T. Galili, dendextend: an R package for visualizing, adjusting and comparing trees of hierarchical clustering. *Bioinformatics* **31**, 3718–3720 (2015).
118. S. R. Harris, SKA: Split Kmer Analysis Toolkit for Bacterial Genomic Epidemiology. *Cold Spring Harbor Laboratory*, 453142 (2018).
